# Hazara virus and Crimean-Congo Hemorrhagic Fever Virus show a different pattern of entry in fully-polarized Caco-2 cell line

**DOI:** 10.1371/journal.pntd.0008863

**Published:** 2020-11-24

**Authors:** Vanessa Monteil, Cristiano Salata, Sofia Appelberg, Ali Mirazimi

**Affiliations:** 1 Department of Laboratory medicine, Karolinska Institutet, Stockholm, Sweden; 2 Department of Molecular Medicine, University of Padova, Padova, Italy; 3 Department of Microbiology, Public Health Agency of Sweden, Solna, Sweden; 4 National Veterinary Institute, Uppsala, Sweden; Saudi Ministry of Health, SAUDI ARABIA

## Abstract

Crimean-Congo Hemorrhagic Fever Virus (CCHFV) and Hazara virus (HAZV) belong to the same viral serotype and family. HAZV has lately been used as a model system and surrogate to CCHFV. However, virus-host cell interaction and level of pathogenicity for these viruses are not well investigated nor compared. In this study, we compared HAZV and CCHFV infection of human polarized epithelial cells to shed light on similarities and differences in virus-host cell interaction between these two viruses. We investigated the pattern of infection of CCHFV and HAZV in fully polarized human cells, the Caco-2 cell line. Polarization of Caco-2 cells lead to difference in expression level and pattern of proteins between the apical and the basolateral membranes. We found that CCHFV virus, in contrast to HAZV, is more likely infecting polarized cells basolaterally. In addition, we found that cytokines/pro-inflammatory factors or other viral factors secreted from CCHFV infected moDC cells enhance the entry of CCHFV contrary to HAZV. We have shown that CCHFV and HAZV early in infection use different strategies for entry. The data presented in this study also highlight the important role of cytokines in CCHFV-host cell interaction.

## Author summary

Crimean-Congo Hemorrhagic Fever virus (CCHFV) is a tick-borne pathogen responsible for a severe acute fever disease in humans, requiring biosafety level 4 laboratory for handling. This is the reason why the molecular pathogenesis of CCHFV remains largely unknown. Hazara virus (HAZV), member of the same serogroup but nor responsible for human disease, is commonly used as surrogate model to study CCHFV in biosafety level 2 laboratory. As an important viral model, it is important to better understand its range of applicability. Using polarized Caco-2 cells, we showed HAZV doesn’t have the same pattern of infection in fully polarized cells than CCHFV. These data were confirmed using compounds able to modulate cell junctions: compounds leaded to opposite effect on respective virus infection capacity. All data together suggest that CCHFV and HAZV receptors have different localization on polarized Caco-2 cells. Moreover, using supernatant of HAZV or CCHFV infected monocyte-derived dendritic cells, we demonstrated that only factors released from CCHFV-infected moDCs are able to enhance CCHFV infection. To our knowledge, this study is the first one showing differences in HAZV and CCHFV entry into polarized target cells and in CCHFV infection modulation by a paracrine effect linked to infected dendritic cells.

## Introduction

Crimean-Congo Hemorrhagic Fever virus (CCHFV) is a tick-borne pathogen responsible for a severe acute fever disease in humans with a fatality rate of 5–30%. Geographic dispersal of CCHFV is primarily in Africa, the Middle East, Asia and Southeast Europe [1], where its main vector, ticks of the *Hyalomma* genus, is endemic [2,3]. Apart from tick-bites, human to human transmission occurs through handling of infected animal blood or tissue. In addition, nosocomial route of CCHFV transmission have been reported in several countries [2–4].

Despite the fact that the disease where described as early as 1944, the molecular pathogenesis of CCHFV remains largely unknown. Others have proposed that microvascular leakage, directly induced by the virus and/or as a result of an overreacting host immune response, cause the hemorrhages seen in CCHF [5]. Several studies have shown that patients with the severe form of disease exhibit a high level of pro-inflammatory cytokines (TNF, Il-6, IFN-ɣ…) [6–11]. Some of these cytokines are known to mediate vascular dysfunction, disseminating intravascular coagulation, organ failure and shock. Interestingly, some *in vitro* studies have confirmed the release of pro-inflammatory cytokines by CCHFV-infected human monocytes-derived dendritic cells (moDCs) [12,13] and human endothelial cells [12,14]. However, the knowledge about the molecular pathogenesis of CCHFV is limited by biological and technical restrictions: i) handling CCHFV requires a biosafety level 4 laboratory (BSL-4), ii) no good *in vitro* or *in vivo* models are available, iii) most of CCHFV cases occur in remote regions where advanced medical structures are lacking.

To get around the problem of BSL-4 laboratory requirement, Hazara virus (HAZV), a tick-borne virus belonging to the same serogroup as CCHFV, has been proposed as a surrogate to CCHFV for *in vitro* and *in vivo* studies [15–17]. HAZV was isolated for the first time 1964 in Pakistan from *Ixodes* ticks [18], and there are no documented cases of human infections reported.

The aim of this study was to set up an *in vitro* model, based on human polarized cells, to investigate the differences in CCHFV and HAZV entry pattern and furthermore explore the effects of cytokines released from infected moDCs on viral entry.

## Material and methods

### Cells, virus and chemicals

Human epithelial colorectal adenocarcinoma cells (Caco-2; kindly provided by Karl-Eric Magnusson, Linköping University) and Vero cells (ATCC) were grown in Dulbecco’s Modified Eagle’s Medium (Thermofisher) supplemented with 1% Non-Essential Amino-Acid (Thermofisher), 10mM Hepes (Thermofisher) and 10% FBS at 37°C, 5%CO_2_. For the transwell experiments, 10^5^ cells per cm^2^ were seeded on a transwell cell culture polycarbonate-treated insert of 0.33cm2 diameter, 3μm-pore (Corning) in a volume of 200μl. The volume in the lower compartment was 600μl. The medium on both sides was changed every other day the confluence of the cell-layer was determined by measuring the transepithelial resistance (TEER) through the transwell membrane using a Millicell-ERS volt-ohmmeter (Millipore, Billerica, MA). The resistance (R), expressed in ohm (Ω), were obtained by subtracting the resistance of an empty membrane from the value of the membrane with growing cells. To obtain a value in Ω/cm^2^, the resistance was multiplied by the surface of the well in cm^2^. To simplify, TEER (Ω/cm^2^) = ((R_cell culture well_ (Ω)–R_blank_(Ω)) x 0.33 (cm^2^). For the experiments investigating viral entry side, cells were grown until indicated time-points. For all the other experiments, cells were grown until the transepithelial resistance reached 500Ω/cm^2^, representing fully polarized cells. CCHFV Ibar10200 strain (produced on SW13 cells, GenBank accession number NC005302) and HAZV strain JC280 (produced on Caco-2 cells, GenBank accession number M86624.1) were used for all experiments.

Phorbol 12-myristate 13-acetate (PMA) and ethylene glycol tetraacetic acid (EGTA) (both Sigma Aldrich) were used at a concentration of 50nM and 15mM respectively.

All incubation steps were performed at 37°C, 5% CO_2_.

### Virus titration

Serial (1:10) dilution of the viruses in DMEM with 2%FBS, were used to infect Vero Cells. Twenty-four hours post infection (hpi) cells were fixed by glacial methanol/aceton (1:1) over-night. The fixed cells were incubated with a CCHFV anti-N antibody (In-house, Agrisera) for 1 hour followed by incubation with Alexa Fluor 488 anti-rabbit antibody (Thermofisher). The CCHFV anti-N antibody cross react with HAZV N protein, allowing also the titration of HAZV. The titers were expressed as Focus-Forming Unit per milliliter (FFU/ml).

### RNA extraction and qRT-PCR

RNA was extracted from cell lysate using Directzole RNA extraction kit (Zymo Research). qRT-PCR was performed using a TaqMan Fast Virus 1-step Master Mix (Thermofisher) and primers for amplification of a segment of HAZV N sequence (Forward: CAAGGCAAGCATTGCACAAC; Reverse: GCTTTCTCTCACCCCTTTTAGGA; Probe: FAM-TGAAGGATGGGTCAAAGA-MGB) or CCHFV N sequence (Forward: CAAGGGGTACCAAGAAAATGAAGAAGGC; Reverse: GCCACAGGGATTGT TCCAAAGCAGAC; Probe 1: FAM-GTCAACACAGCAGGGTGCATGTAG AT-MGB; Probe 2: FAM-TGTAAGCACGGCAGGGTGCATGTAAAT-MGB; Probe 3: FAM-ACTCCAATGAA GTGGGGGAAGAAGCT-MGB) on a Capillaries LightCycler (Roche) using RNase P RNA levels for normalization.

### Infection of polarized cells

Caco-2 cells grown on transwell membrane were infected with HAZV or CCHFV either apically (in the well) or basolaterally (inverting the well) at a multiplicity of infection (MOI) of 1 in a final volume of 100μl of culture medium with 2% FBS for 1 hour. Then, the well were washed 3 times with PBS and cultured during 24 hours in fresh complete culture medium (200μl apically and 600μl basolaterally). Twenty-four hours later, supernatants were removed, wells washed 3 times on both sides with PBS and thereafter 300μl of Trizol (Thermofisher) per well were used to lyze the cells. HAZV and CCHFV infection experiment were performed in duplicate in two independent experiments.

### Isolation, differentiation and infection of monocyte-derived dendritic cells

Peripheral blood mononuclear cells (PBMCs) were isolated from human healthy blood donors (buffy coat) by ficoll separation. The ethical committee at the Karolinska Institutet approved our studies involving human samples. CD14^+^ cells were positively selected using anti-CD14^+^ MACS beads (Miltenyi Biotec) according to manufacturer instructions. Following separation, a sample was stained for CD14 (Thermofisher) and checked for purity by flow cytometry using a FACS Canto II (BD Biosciences). Results were analyzed using the Flowjo software (LLC).

Monocyte-derived dendritic cells (moDCs) were obtained by culturing the purified monocytes in a 6-well plate at 4.10^6^ cells per well in 5ml of RMPI medium (Thermofisher) supplemented with 10% FBS, 75ng/ml of recombinant human GM-CSF (Peprotech) and 50ng/ml of human recombinant IL-4 (Peprotech). On day 3, half of the medium was removed replaced by fresh medium containing the same concentration of cytokines as previously. On day 6, a cell sample were stained and tested for expression of CD14 (Thermofisher), HLA-DR (Thermofisher) and CD1a (Thermofisher) surface proteins using flow cytometry (FACS Canto II, BD Biosciences) to confirm the immature dendritic cells phenotype.

Four million cells per well were infected with HAZV or CCHFV at a MOI of 0.1 in 1ml of RPMI medium supplemented with 2% FBS during 1 hour at 37°C, 5% CO_2_. After infection, the inoculum was carefully removed and 5ml of RPMI containing 10% FBS added. Twenty-four, 48 and 72 hpi, cell supernatants were collected, the cells were recovered, washed 3 times with PBS and then lysed using 300μl of Trizol per well. Viral RNA levels in each sample was determined by qRT-PCR as described above.

### Effect of supernatant from infected moDCs on virus infection of polarized cells

Supernatants from HAZV, CCHFV or mock-infected moDCs were collected at appropriate time points based on maximum cytokine release (3 days post-infection for HAZV and 2 days post-infection for CCHFV) (S1 Fig) and UV-treated for 5 minutes to inactivate virus present in the samples (see below). HAZV or CCHFV virus was added to the treated supernatants (final volume of 100μl) before infecting polarized Caco-2 cells on transwell at an MOI of 1, either apically or basolaterally, for 1 hour. During the infection, the other side of the transwell was treated with 100μl (apically) or 600μl (basolaterally) of complete DMEM medium. Thereafter, the inoculums was removed, cells were washed 3 times with PBS on both sides and new complete DMEM 10% FBS medium was added on both sides (200μl in the apical compartment, 600μl in the basolateral compartment). Twenty-four hpi, supernatants were removed, cells were washed 3 times with PBS on both sides and then lysed using 300μl of Trizol per well. Viral RNA levels in each sample was determined by qRT-PCR as described above. For each donor, the fold change in viral RNA was expressed by comparing cells infected with supernatant from HAZV or CCHFV infected moDCs to control cells. Control cells being cells infected with supernatant from mock-infected moDCs.

### Compound treatment

Fully polarized Caco-2 cells cultured on transwell membrane were treated apically only with 50nM of Phorbol 12-myristate 13-acetate (PMA) for 1 hour before infection or, after PMA removal, also treated with 15mM of ethylene glycol tetraacetic acid (EGTA). After treatment, compounds were removed and cells were infected apically with HAZV or CCHFV at a MOI of 1 in 1 ml culture medium without FBS for 1 hour. After infection, cells were washed 3 times with PBS on both sides and complete culture medium was added as described above. Twenty-four hpi, cells were washed 3 times with PBS on both sides then 300μl of Trizol (Thermofisher) per well were used for cells lysis.

### ELISA

Interleukin (IL)-6, IL-8, IL-10, Tumor necrosis factor (TNF)-α and Interferon (IFN)-γ levels were measured using specific ELISA kits (Mabtech AB) following the company instructions. The plates were read at 650nm on BioTek Synergy HT instrument.

### CCHFV UV-inactivation control

The CCHFV Ibar10200 stock was placed in one well of two 6 well plates (500ul/well). One plate was left untreated (mock), the other was submitted to UV-irradiation for 5 min (UV mineral light lamp, model UVG-54; 254 nm; UVP, Upland, CA). One hundred μl of mock-treated or UV-treated samples were mixed 1:1 with Leibovitz medium containing 5% FBS (Thermofisher) and the obtained 200ul were used to infect SW13 cells. Cells were incubated for 48h (37°C, 5% CO_2_) and thereafter fixed in ice cold methanol/aceton (1:1) at -20°C for 24h. Cells were stained first with a rabbit anti-CCHFV NP antibody (in house, 1:250) and secondly with an Alexa Fluor Plus 488 anti-rabbit antibody (Thermofisher, 1:1000) and DAPI (Roche, 1:1000) (S2 Fig).

## Results

### CCHFV and HAZV exhibit a different entry pattern in polarized cells

**Fig 1:**
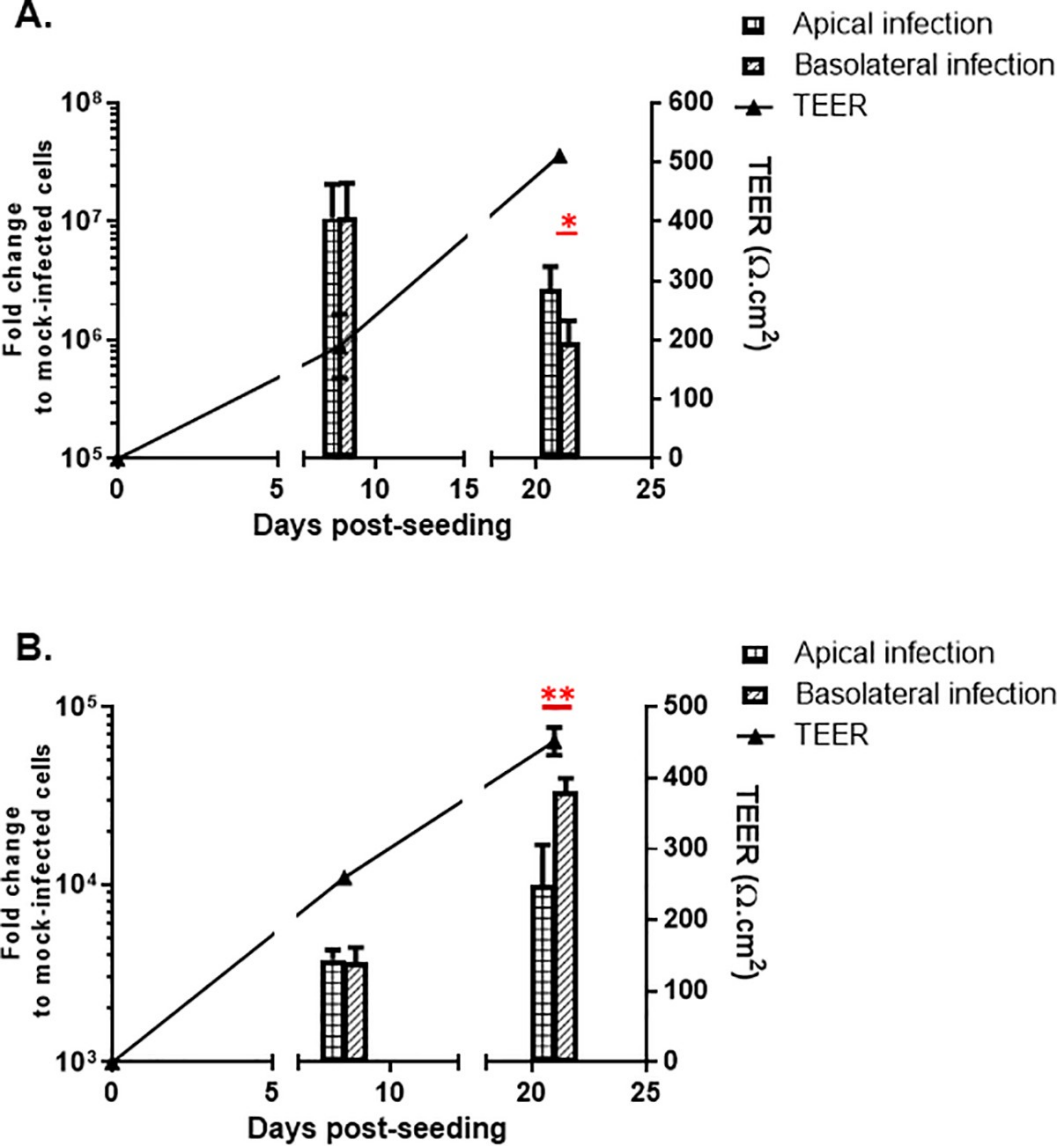
Virus entry in Caco-2 cells. Caco-2 cells were infected apically or basolaterally at different stages of polarization with (A) HAZV or (B) CCHFV. Data show the relative variation of viral RNA in comparison to control (mock). Bars represent mean ±SD based on three independent experiments. Statistical analysis: student t-test (*: p<0.05; **: p<0.01). Plaid bands: Apical Infection. Oblique bands: Basolateral infection.

Previously, we have shown that CCHFV are more likely to entrer and release on the basolateral side of highly polarized Madin-Darby Canine Kidney cell line (MDCK-1)[19]. In the present study, we have established a more relevant *in vitro* model system based on human Caco-2 cells. To investigate the pattern of HAZV infection on polarized cells compared to CCHFV infection, we infected human polarized cells (Caco-2) at different stages of polarizations with HAZV or CCHFV. As shown in Fig 1A, the level of polarization of the cells influences the pattern of HAZV infection. Interestingly, this data shows that HAZV can infect fully polarized cells on both sides but with a preference for the apical side. As previously described, using MDCK-1 polarized cells [19], we show here that CCHFV infects the Caco-2 polarized cells mainly on the basolateral side (Fig 1B) indicating a difference between HAZV and CCHFV entry strategy.

**Fig 2:**
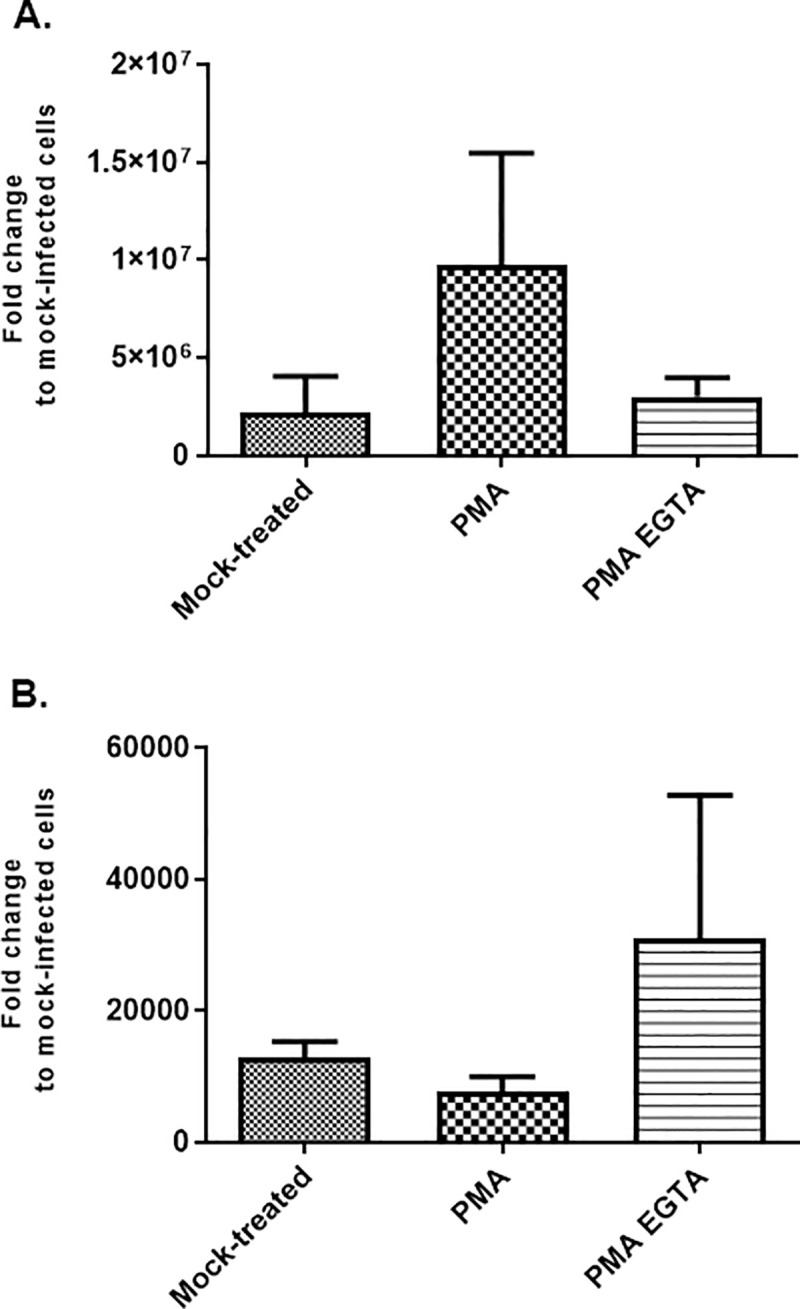
Effect of PMA or PMA+EGTA on virus entry. Fully polarized Caco-2 cells were treated apically with PMA only or with PMA and then EGTA before infection with (A) HAZV or (B) CCHFV at MOI 1. Data show the relative variation of viral RNA in comparison to control (mock). Bars represent mean ±SD based on three independent experiments.

To further investigate the difference in the side of infection between HAZV and CCHFV, we used compounds affecting the cell junctions: PMA, stabilizing the tight-junctions in intestinal cells [20], makes all the intercellular junctions Ca^2+^ dependent, or EGTA dissociated only the Ca^2+^ dependent junctions. The use of PMA followed by EGTA leads to opening of all cell junctions. The polarized cells were treated with PMA only or with PMA and EGTA. Then the cells were infected apically either with HAZV or CCHFV. As shown in Fig 2A, we found that PMA treatment of polarized cells increases the apically HAZV infection withroughly one log compared to mock-treated cells. When PMA treatment is followed by EGTA treatment for 1 hour before infection with HAZV, the level of infection decreases compare to PMA treatment only (Fig 2A). Interestingly, in contrast to HAZV, PMA followed by EGTA treatment increases CCHFV infection apically compared to only PMA treatment (Fig 2B).

### Factors released by CCHFV infected moDCs improve CCHFV basolateral infection

**Fig 3:**
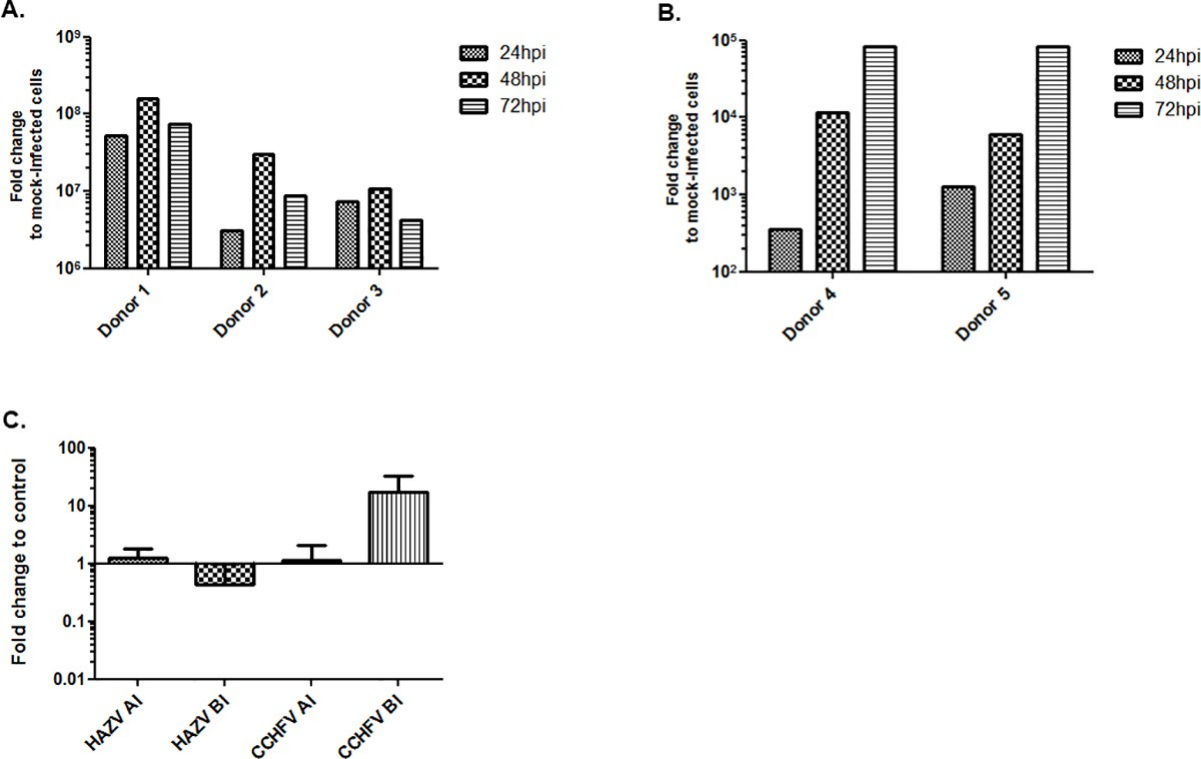
Effect of supernatant from infected moDCs on virus infection. Level of viral RNA in (A) HAZV or (B) CCHFV infected moDCs at 24, 48 and 72h pi. (C) Effect of molecules (cytokines/proinflamatory factors/viral factor) released by infected moDCs on apical (AI) or basolateral (BI) infection with HAZV (left) or CCHFV (right). Results indicate the relative yield of intracellular viral RNA in comparison to control cells (infection in the presence of supernatant collected from mock-infected moDCs). Bars mean ±SD based on three independent experiments.

To investigate if the inflammatory response from infected moDCs may influence the pattern of apical/basolateral infection of HAZV or CCHFV, monocytes from 5 healthy donors were differentiated into moDCs and infected with HAZV or CCHFV. The analysis of viral RNA inside moDCs showed a similar level of infection between donors for both viruses (Fig 3A and 3B), except the moDCs isolated from donor 1 that showed a higher level of HAZV RNA compare to the other donors (Fig 3A).

Polarized Caco-2 cells were infected apically or basolaterally with HAZV or CCHFV in the presence of supernatant from infected moDC with corresponding virus. To inactivate the virus produced by infected moDCs, supernatants were UV-treated before use (S2 Fig). Supernatant from HAZV-infected moDCs did not lead to significant changes on the level of apical infection, while the level of basolateral infection decreased compared to control, (Fig 3C). Most interestingly, supernatant from CCHFV-infected moDCs increased the level of basolateral infection over one log compared to control, while no effect was observed on apical infection (Fig 3C).

**Fig 4:**
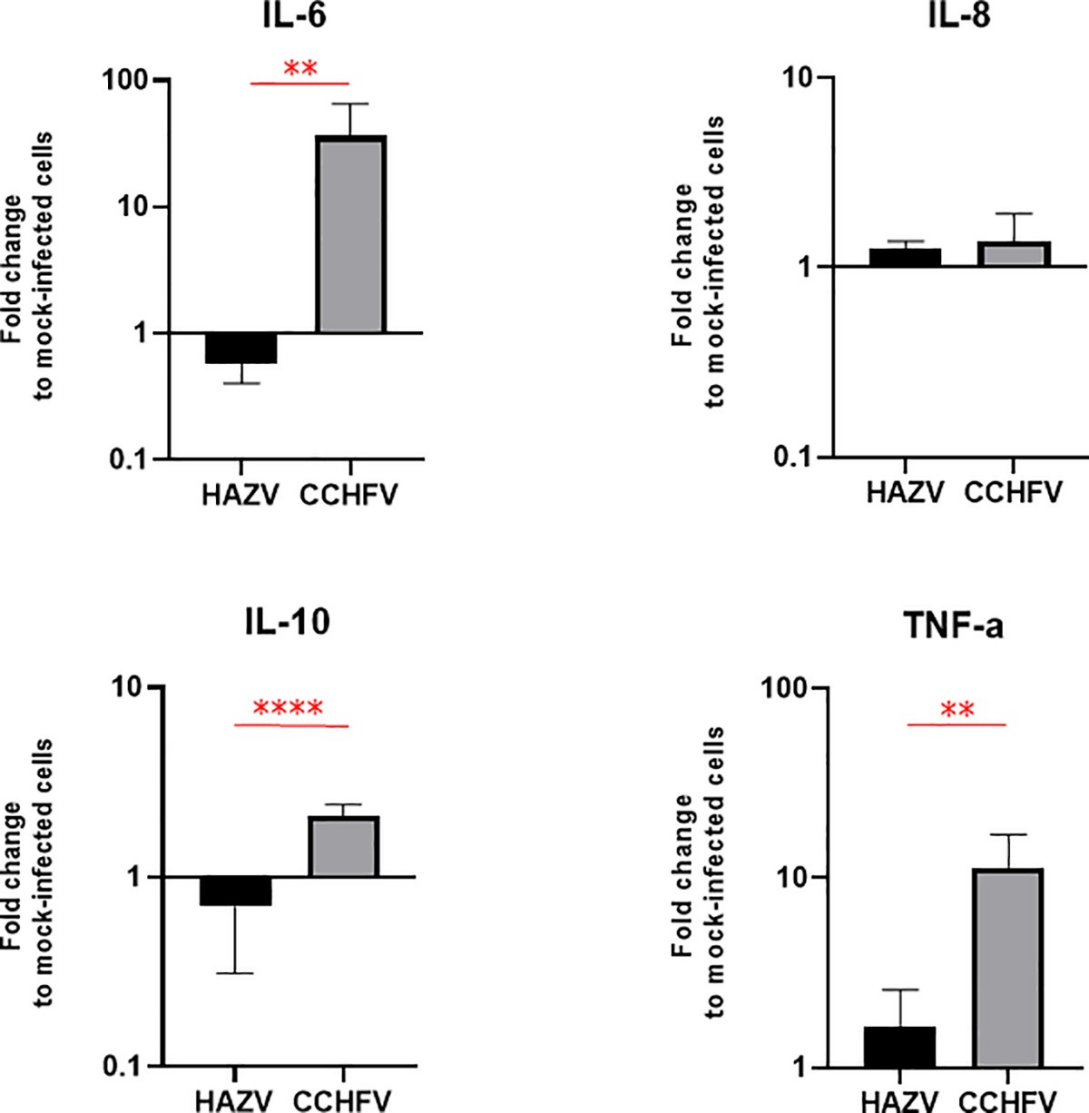
Detection of selected cytokines in supernatant from HAZV- or CCHFV-infected moDCs. IFN-γ was not detected. Bars represent mean ±SD based on three replicates. Statistical analysis: student t-test (**: p<0.01, ****: p<0.0001).

Previously, we have demonstrated that *in vitro* infection of moDCs with CCHFV induced secretion of cytokines involved in inflammation [12,14]. Thus, we decided to compare levels of IL-6, IFN-γ, TNF-α, IL-8 and IL-10 in supernatant from HAZV- or CCHFV-infected moDCs. In supernatant from CCHFV infected moDCs, IL-6, IL-10 and TNF-α levels increased compared to control, while they did not in supernatants from HAZV-infected moDCs. Hence, there were significantly higher levels of IL-6, IL-10 and TNF-α in supernatant from CCHFV infected moDC compared to in supernatant from HAZV infected moDCs (Fig 4). No difference in IL-8 production between CCHFV- and HAZV-infection was observed. IFN-γ was not detected at all in the tested conditions.

## Discussion

One of the most obvious symptoms in CCHF patients is bleeding at different body sites. An increased permeability of the endothelium or cellular detachment from underlying tissues could be the explanation of this clinical sign. Viral tropism is a major determinant of pathogenesis, which refers to the distribution of viral receptors in the organism. Identification of viral receptors and regulation of their expression in different tissues and organs contributes to the understanding of the molecular pathogenesis of a virus. To facilitate and enable research on this topic, scientist use different model systems based on viruses with similar pathogenicity.

Commonly, to study highly pathogenic viruses surrogate models are used, such as viruses from the same family, like Pichinde virus [21] and Lymphocytic Choriomeningitis virus [22] are used to study Lassa virus. To date, HAZV which together with the recently discovered Tofla virus [23], belong to the same serogroup as CCHFV is used as a surrogate for CCHFV infections both *in vitro* and *in vivo*. In fact, HAZV was shown to, just like CCHFV [24], to establish lethal infection in IFNα/βR^-/-^ mice [15]. Although some studies have shown that the same type of viral proteins establish similar interactions/effects with cellular proteins for both viruses [25,26], further researches are needed to compare the biology of HAZV and CCHFV.

To investigate the early steps of infection, we have used an *in vitro* model system based on highly polarized human epithelial cells. Epithelial cells have asymmetric plasma membranes containing distinct apical and basolateral domains with differences in the expression/distribution of cellular proteins (including the viral receptors), which affect viral entry.

We have previously shown, by using a highly polarized MDCK-1 canine cells model, that CCHFV has a preferential basolateral entry and release [19]. However, MDCK-1 is not the most appropriate model to study the molecular pathogenesis of CCHFV since dogs are genetically distant from human and no clinical signs of CCHF have ever been described in infected-dogs. In this study, we aimed to set up a more relevant *in vitro* cell culture system for CCHFV to i) compare the entry site for CCHFV and its non-pathogenic surrogate HAZV and ii) investigate the paracrine role of cytokines released from infected moDC on the early steps of infection.

Caco-2 is a human epithelial cell line, which becomes polarized when grown on permeable membranes. This model is mainly used to study the cellular permeability/absorption of compounds and was also used to study the entry of Ebola virus [27], Hepatitis C virus [28], Poliovirus [29], and Rotavirus [30]. Polarization of human epithelial cells leads to a different pattern of surface proteins on the apical side compared to the basolateral side. Caco-2 cells polarization on a semi-permeable filter is a complex event with cellular genes modulation [31] leading more particularly to the development of tight-junction and desmosomes, and the rearrangement of surface proteins. Here, we have shown that the level of polarization affects which side the two different viruses will most likely use for entry (Fig 1), as it could impact the localization of the viral receptor. An explanation to the increase in CCHFV infection observed during polarization could be that polarization leads to an enhanced expression of CCHFV entry receptor(s). In contrast, polarization of the cells could decrease the expression of HAZV entry receptor(s) and thus, explain the decrease in HAZV infection during the polarization process (Fig 1). Although HAZV and CCHFV belong to the same serogroup, they don’t have the same pattern of infection in fully polarized cells, suggesting the use of different receptors, at least in Caco-2 cells. In addition, we have to highlight that HAZV has a much faster replication process in Caco-2 compared to CCHFV.

To further investigate the difference in entry mode of both viruses, fully polarized Caco-2 cells were treated with only PMA or with PMA and EGTA before infection. PMA, that make the intercellular junctions Ca^2+^ dependent, was shown to stabilize the junctions in intestinal cells [20]. Treatment with EGTA, a Ca^2+^ chelator, after the treatment with PMA, leads to complete opening of intercellular junctions and disrupting of the polarization of treated cells. PMA treated cells show more sensibility/permissiveness to HAZV infection apically compared to mock-treated cells. This is most probably due to more access to the viral receptor on the apical side, as PMA treated Caco-2 cells have tighten junctional barriers and thereby reduce mobility of proteins around apical and basolateral side. In opposite, we found that the level of infection of CCHFV from apical side is reduced when we tightened the junctions (using PMA) compared to non-treated cells. The opening of all junctions (PMA and EGTA) increases the level of infection of CCHFV compared to the non-treated and only PMA-treated cells, which suggest that most of the receptors/co receptors of CCHFV are localized on the basolateral side of polarized cells. All data together suggest that CCHFV and HAZV receptors have different localization on polarized Caco-2 cells.

Previous studies have suggested that the disease progression of viral hemorrhagic fevers could be due to a cytokine storm and high viremia [32,33]. We have previously showed that pro-inflammatory cytokines secreted from the CCHFV-infected monocyte-derived dendritic cells (moDCs) activate endothelial cells and lead an overexpression of ICAM-1, VCAM-1 and E-selectin, suggesting an indirect modulation of endothelial cell junctions [14]. Based on these data and the above reported results showing a different pattern of entry of HAZV and CCHFV, we investigated the effect of factors released by infected moDCs on the infection pattern in highly polarized cells. Lutschg V. et al showed that interleukin-8 released by infected macrophages could reverse the localization of the receptor for adenovirus from the apical side to the basolateral side [34]. We have found that factors secreted from HAZV infected moDCs lead to less basolateral infection level and has no effect on apical infection (Fig 3C). In contrast, factors released by CCHFV infected moDCs lead to a strong increase in the level of infection on basolateral side (Fig 3C), which may indicate more accumulation of receptor or co-receptors of CCHFV at basolateral side, or an up-regulation of their expression. But contrary to what has been observed for adenoviruses, IL-8 seems not be responsible for any receptor switching between apical / basolateral side or other effects on viral entry. In fact, the level of released IL-8 by HAZV and CCHFV infected moDCs are similar (Fig 4). Due to the number of cells isolated from each patient sample, we were unable to use cells from the same donor to test simultaneously both viruses but, as showed in Fig 3, the viral replication pattern is constant for each virus supporting that there is not a donor effect.

CCHF disease severity seems to be correlated with a high level of pro-inflammatory cytokines [6,9], partly released by dendritic cells and macrophages [12,13]. Accordingly, we showed that only CCHFV infection of moDCs induced a significant production of IL-6, IL-10 and TNF-α in comparison to the non-pathogenic HAZV (Fig 4). Several studies have shown that patients with the severe form of disease exhibit high levels of IL-6 and TNF-α [7,9], or only a high level of TNF-α [35], while the role of IL-10 in viral pathogenesis is still debated between studies [7,36]. Interestingly, we demonstrated that factors released from CCHFV-infected moDCs are able to enhance CCHFV infection in polarized cells. Even if this observation doesn’t allow to discriminate the effect of cytokines from the effect of viral factors, this mechanism could be involved in the higher viremia and endothelial damage observed in the severe CCHF cases [10,33].

To our knowledge, this study is the first one showing differences in HAZV and CCHFV entry into polarized target cells and in CCHFV infection modulation by a paracrine effect linked to infected dendritic cells. To further support our observation, studies using CCHFV clinical isolates instead of the CCHFV laboratory strain Ibar10200 used in this study has to be conducted. However, our data must be considered in future studies using HAZV as a model for CCHFV entry and pathogenesis to better understand the range of applicability of this important viral model.

## Supporting information

S1 Fig Cytokines detection in HAZV infected moDCs supernatant. 2dpi sample shows the higher amount/kind of cytokines released corresponding to the higher level of viral RNA in cells showed in Fig 3A.
(TIF)

S2 Fig UV-inactivation of CCHFV. CCHFV stock was UV-treated or mock-treated during 5min and used to infect SW13 cells. Cells were fixed 48hpi. UV-treatment completely inactivate CCHFV.
(TIF)
