# Supplementary figures and images for: Hazara virus and Crimean-Congo Hemorrhagic Fever Virus show a different pattern of entry in fully-polarized Caco-2 cell line
Source: PLoS Negl Trop Dis. 2020 Nov 24;14(11):e0008863. doi: 10.1371/journal.pntd.0008863 (PMC7723249; doi:10.1371/journal.pntd.0008863)

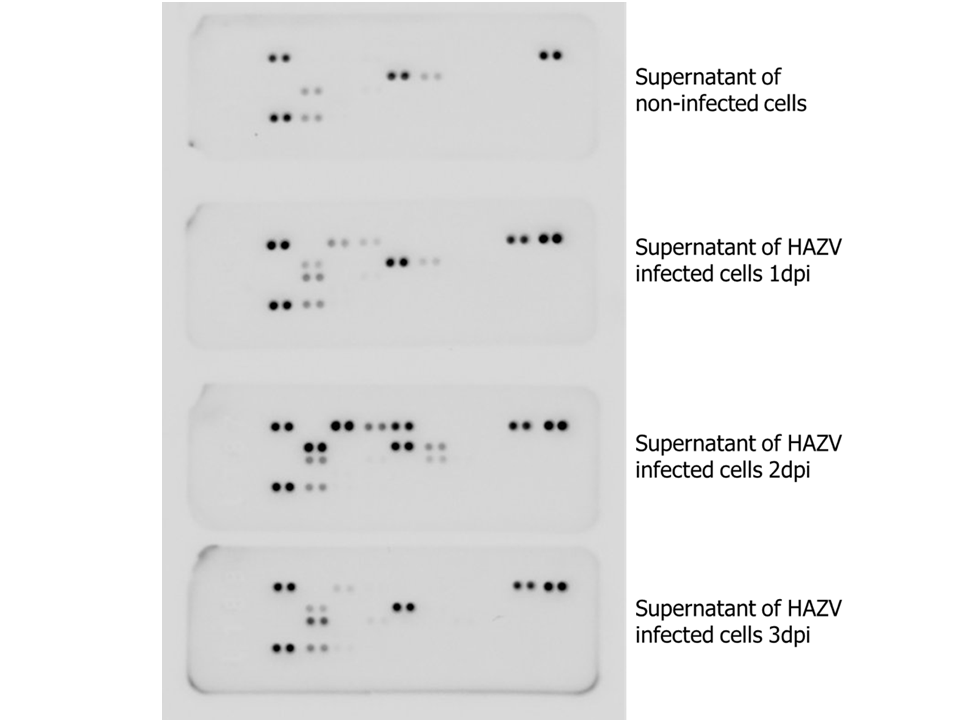

Supplement: S1 Fig — 2dpi sample shows the higher amount/kind of cytokines released corresponding to the higher level of viral RNA in cells showed in Fig 3A. (TIF) [file pntd.0008863.s001.tif]

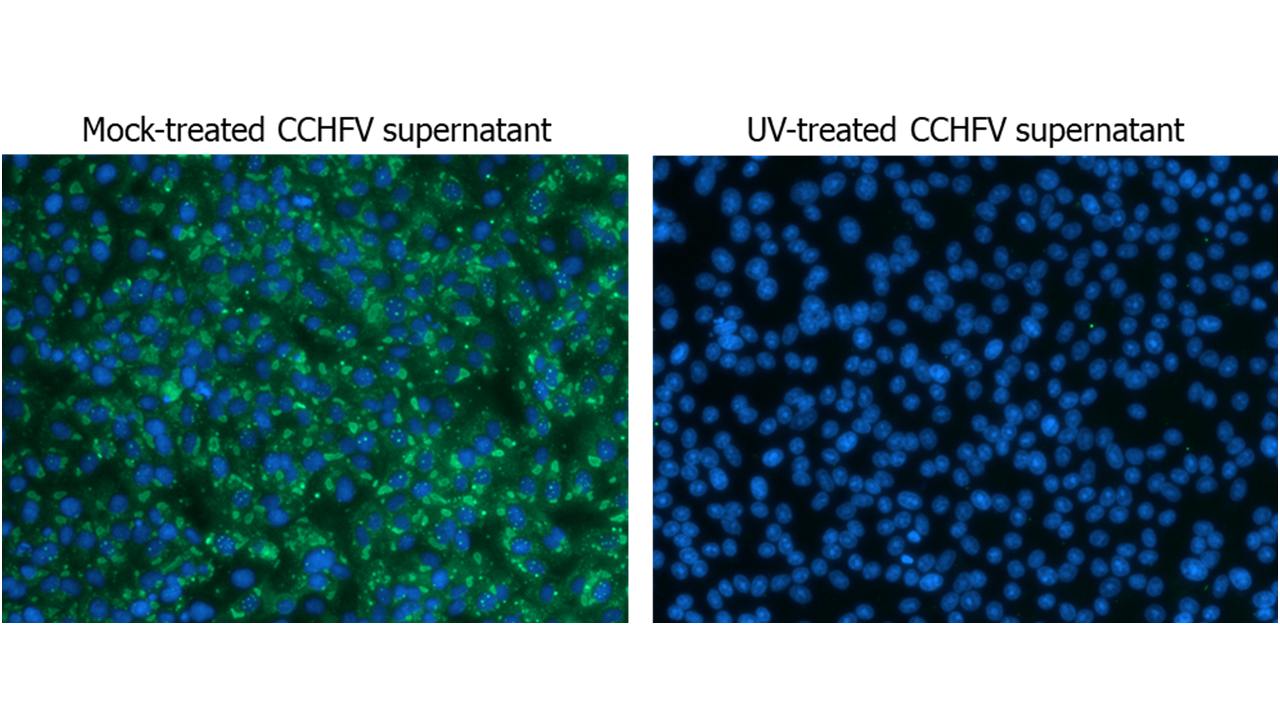

Supplement: S2 Fig — CCHFV stock was UV-treated or mock-treated during 5min and used to infect SW13 cells. Cells were fixed 48hpi. UV-treatment completely inactivate CCHFV. (TIF) [file pntd.0008863.s002.tif]
